# Inverted urothelial papilloma of the upper urinary tract: description of two cases with systematic literature review

**DOI:** 10.1186/s13000-020-00961-9

**Published:** 2020-04-22

**Authors:** R. Santi, I. C. Galli, V. Canzonieri, J. I. Lopez, G. Nesi

**Affiliations:** 1 Pathology Unit, Careggi University Hospital, Florence, Italy; 2 Pathology Section, Department of Health Sciences, University of Florence, Viale Pieraccini 6, 50139 Florence, Italy; 3 Centro di Riferimento Oncologico di Aviano (CRO), IRCCS, Aviano, Italy; 4 Department of Medical, Surgical and Health Sciences, University of Trieste Medical School, Trieste, Italy; 5 University of the Basque Country, Barakaldo, Spain

**Keywords:** Inverted urothelial papilloma, Upper urinary tract, Molecular markers, Microsatellite instability

## Abstract

**Background:**

Inverted urothelial papilloma (IUP) of the upper urinary tract is an uncommon benign tumour that occasionally presents as a polypoid mass causing urinary obstruction. Histologically, IUP is characterised by a proliferating urothelium arranged in cords and trabeculae, in continuity with overlying intact epithelium, and extending into the lamina propria in a non-invasive, endophytic manner. Cytological atypia is minimal or absent. Top differential diagnoses include urothelial carcinoma with inverted growth pattern and florid ureteritis cystica. Although urothelial carcinomas of the upper urinary tract with prominent inverted growth pattern commonly harbour microsatellite instability, the role of the mutator phenotype pathway in IUP development is still unclear. The aim of this study was to describe two additional cases of IUP of the upper urinary tract, along with an extensive literature review.

**Case presentation:**

We observed two polypoid tumours originating in the renal pelvis and the distal ureter, respectively. Both patients, a 76-year-old woman and a 56-year-old man, underwent surgery because of the increased likelihood of malignancy. Histology was consistent with IUP and patients are alive and asymptomatic after long-term follow-up (6 years for the renal pelvis lesion and 5 years for the ureter lesion). The tumours retained the expression of the mismatch-repair protein MLH1, MSH2, and PMS2 whereas loss of MSH6 was found in both cases.

**Conclusions:**

When completely resected, IUP does not require rigorous surveillance protocols, such as those for urothelial carcinoma and exophytic urothelial papilloma. It is therefore important for the surgical pathologist to be aware of this rare entity in order to ensure correct patient management.

## Background

Inverted urothelial papilloma (IUP) is a rare lesion, histologically similar to inverted papilloma of the nasal cavity and paranasal sinuses. First reported in 1927 by Paschkis as “polypoid adenoma of the bladder” [1], it was later described in 1963 by Potts and Hirst as a distinct tumour entity of the urinary bladder [2]. IUP accounts for approximately 2% of all urothelial neoplasms. It usually occurs at the bladder neck, trigone or prostatic urethra, but is rare in the upper urinary tract. To the best of our knowledge, 68 IUP cases of the renal pelvis and ureter have been described in the English literature (Tables 1 and 2) [3–52].

**Table 1:** IUP of the renal pelvis (RP) previously reported in the English Literature (NS = Not Stated; NA = Not Assessed)

Reference	Age	Sex	Presentation	Site	Gross/Maximum Diameter (cm)	Associated Urothelial Lesions	Treatment	Recurrence (Follow-Up)
*Matz* et al. *(1974)* [3]	68	M	Haematuria, flank pain	Left RP	Nodule/1.5	None	Nephroureterectomy	None (2 ys)
*Assor (1976)* [4]	79	M	Haematuria, flank discomfort	Right RP	Sessile polyp/1.5	None	Partial resection	NS
*Cameron* et al. *(1976)* [5]	58	F	NS	RP (side NS)	NS/3	None	Nephroureterectomy	Patient died of carcinoma of the endometrium four years later
*Di Cello* et al. *(1980)* [6]	53	M	Asymptomatic	Left uretero-pelvic junction	Sessile polyp/3	None	Nephroureterectomy	NS
*Theoret* et al. *(1980)* [7]	89	M	Asymptomatic (autopsy finding)	RP (side NS)	NS	None	NA	NA
*Uyama* et al. *(1981)* [8]	73	M	Haematuria	Left RP	NS/2.5	None	Nephroureterectomy, radiation and chemotherapy	None (5 ys)
*Anderström* et al. *(1982)* [9]	62	M	Asymptomatic	Left RP	Nodule/3	Synchronous grade 2 transitional cell carcinoma of contralateral RP and non-invasive grade 2 transitional cell carcinoma of the bladder; history of recurrent grade 2 transitional cell carcinoma of the bladder	Extracorporeal resection of ureter and RP and autotransplant of kidney to bladder	Patient died of metastatic poorly differentiated squamous cell carcinoma of the bladder three years later; no recurrence in the kidney where IUP was diagnosed
*Anderström* et al. *(1982)* [9]	49	M	Ureteral colic	Right RP	Nodule/NS	None	NS	NS
*Watters* et al. *(1983)* [10]	65	M	Haematuria	Left RP	Pedunculated polyp/1	None	Nephroureterectomy	NS
*Lausten* et al. *(1984)* [11]	63	M	NS	Right RP	Pedunculated polyp/1	Grade 3 invasive polypoid transitional cell carcinoma in the contralateral RP after 8 years	Nephrectomy	None (8.5 ys)
*Taylor* et al. *(1986)* [12]	65	M	Haematuria	Right RP	Sessile polyp/NS	None	Nephroureterectomy	None (2 ys)
*Schulze* et al. *(1986)* [13]	53	M	Haematuria	Right RP	Sessile polyp/2.5	None	Nephroureterectomy	NS
*Schulze* et al. *(1986)* [13]	55	M	Haematuria	Left RP and ureter	Not apparent at gross examination	None	Nephrectomy	NS
*Romanelli (1986)* [14]	52	M	Haematuria, renal colic,	Right RP	Sessile polyp/2.1	None	Nephroureterectomy	NS
*Yamaguchi* et al. *(1988)* [15]	73	M	Haematuria	Left RP	Pedunculated polyp/0.6	Synchronous low grade transitional cell carcinoma of the bladder (ureteral orifice)	Nephrectomy	None (1 y)
*Schultz* et al. *(1988)* [16]	58	M	Haematuria	Left RP	NS	Synchronous superficial grade 2 transitional cell carcinoma of the contralateral ureter (nephroureterectomy with excision of the bladder cuff)	Pyelotomy and endoscopic resection	IUP of the bladder 1 y later
*Aubert* et al. *(1988)* [17]	34	M	Haematuria	Left RP	NS	None	Nephroureterectomy	None (18 months)
*Kyriakos* et al. *(1989)* [18]	73	F	Asymptomatic	Multiple lesions: Junction between a upper pole major calyx and right RP (I); right calix (II); distal right ureter (III and IV)	Polyp/2.6 (I); slightly elevated nodule/1 (II); polyp/0.5 (III); polyp/1.2 (IV)	None	Nephroureterectomy	None (11 months)
*Bagley* et al. *(1990)* [19]	64	M	Haematuria	Right RP	Nodule/1	Recurrent transitional cell carcinoma of the bladder	Ureteropyeloscopy with endoscopic resection	None (6 months)
*Bassi* et al. *(1991)* [20]	51	M	Haematuria, flank pain	Left RP	Sessile polyp/0.5	None	Partial resection	NS
*Vlassopopulos* et al. *(1992)* [21]	59	M	Haematuria, flank pain	Left RP	Sessile polyp/2	None	Nephroureterectomy	None (12 months)
*Ueda T* et al. *(1992)* [22]	71	M	Asymptomatic	Right RP	Nodule/4	None	Nephrectomy	Synchronous clear cell carcinoma of the homolateral kidney, treated with surgery and anticancer drugs. No recurrence from IUP (21 months)
*Spevack* et al. *(1995)* [23]	64	M	Haematuria	Right RP	Pedunculated polyp/2.5	None	Partial resection	None (42 months)
*Chiura* et al. *(1998)* [24]	63	M	Haematuria	Right RP	NS/3	Transitional cell carcinoma of the left distal ureter three years later, treated with surgery, radiation therapy and chemotherapy	Nephroureterectomy	None (1 y after surgery for carcinoma)
*Chiura* et al. *(1998)* [24]	53	M	Haematuria	RP	NS	Pyelitis cystica	Nephroureterectomy	NS
*Chiura* et al. *(1998)* [24]	64	M	Asymptomatic	Right RP	NS	Recurrent transitional cell carcinoma of the bladder (previous and subsequent to IUP diagnosis)	Ureteroscopy and biopsy	Transitional cell carcinoma in the homolateral kidney and ureter 9 ys later
*Darras* et al. *(2005)* [25]	52	M	Haematuria, occasional discomfort in the lower abdomen	Left RP	Polyp/NS	Synchronous IUP of the bladder	Partial resection	None (NS)
*Luo* et al. *(2012)* [26]	62	M	Asymptomatic	Right RP	Pedunculated polyp	None	Nephroureterectomy	None (NS)
*Luo* et al. *(2012)* [26]	66	M	Haematuria	Left RP	Pedunculated polyp	None	Nephroureterectomy	None (NS)
*Luo* et al. *(2012)* [26]	64	M	Haematuria	Left RP	Pedunculated polyp	None	Nephroureterectomy	None (NS)
*Luo* et al. *(2012)* [26]	73	F	Flank pain	Right RP	Pedunculated polyp	None	Nephroureterectomy	None (NS)

**Table 2:** IUP of the ureter (U) previously reported in the English Literature (NS = Not Stated; NA = Not Assessed)

Reference	Age	Sex	Presentation	Site	Gross/Maximum Diameter (cm)	Associated Urothelial Lesions	Treatment	Rrecurrence (Follow-Up)
*Geisler* et al. *(1980)* [27]	77	M	Flank pain	Left middle U	Pedunculated/2.5	None	NephroUectomy	NS
*Silverstein* et al. *(1981)* [28]	65	M	Asymptomatic	Left middle U	Pedunculated/2.5	None	Partial resection	NS
*Silverstein* et al. *(1981)* [28]	68	M	Haematuria	Right middle U	Polypoid/2.5	None	NephroUectomy	NS
*Fromowitz* et al. *(1981)* [29]	75	M	Haematuria	Right U, at junction of proximal and middle thirds	Flat, polypoid/1.8	None	NephroUectomy	NS
*Fromowitz* et al. *(1981)* [29]	56	M	Asymptomatic	Right distal U	Raised/1.1	Adenocarcinoma of the bladder 7 months later with three recurrences during next 2 ys	NephroUectomy	None (2 ys)
*Ajrawat* et al. *(1982)* [30]	86	F	Flank pain	Right distal U	Lobulated mass/1.5	None	Partial resection	NS
*Naito* et al. *(1983)* [31]	68	M	Haematuria	Right distal U	Pedunculated/1.5	None	NephroUectomy	None (2 ys)
*Jacobellis* et al. *(1983)* [32]	59	F	Haematuria, flank pain	Left lumbar U	Sessile/3	Synchronous conventional papilloma of homolateral lower calix	NephroUectomy	NS
*Embon* et al. *(1984)* [33]	69	M	Haematuria	Right distal U	Polypoid/3	None	Partial resection	None (9 months)
*Lausten* et al. *(1984)* [11]	60	M	Asymptomatic	Right proximal U	Sessile tumour/ 0.3	Grade 2 non-invasive transitional cell papilloma located above the homolateral Uic orifice 1 and half years earlier	Cranial heminephroUectomy	None (19 months)
*Lausten* et al. *(1984)* [11]	71	M	Flank pain (prostatism)	Right proximal U	Pedunculated tumour/ 1	None	Partial U resection	None (18 months)
*Perrin* et al. *(1984)* [34]	63	M	Haematuria, renal colic	Left middle U	Polypoid/NS	None	Partial resection	Dead after 2 ys of cirrhosis; no recurrence of Ual lesion
*Mottola* et al. *(1984)* [35]	56	M	Haematuria, flank pain	Right lumbar U	NS	None	Partial resection	None (12 months)
*Palvio (1985)* [36]	50	M	Haematuria	Distal portion of the left U (above the Ual orifice)	Pedunculated tumour/ NS	After 8 ys from the first diagnosis of IUP of the distal U, the patient underwent nephroUectomy for two lesions at the Uopelvic junction and in the distal part of the U (IUP with areas of non-invasive transitional cell carcinoma, grade 2)	TUR	Yes, after 3 ys
*Moss* et al. *(1987)* [37]	79	M	Asymptomatic	Right U	NS/1	None	U resection during hemicolectomy	None (3 months)
*Corkill* et al. *(1987)* [38]	62	M	Haematuria	Left distal U	Polypoid/0.8	None	Partial resection	None (7ys)
*Duchek* et al. *(1987)* [39]	24	M	Haematuria, renal colic	Right middle U	Pedunculated lesion/NS	None	Local resection	None (5 ys)
*Abulafi A* et al. *(1987)* [40]	62	M	Haematuria	Right proximal U	Pedunculated lesion/NS	None	Local resection	NS
*Villani U* et al. *(1987)* [41]	56	M	Haematuria	Left pelvic U	NS	Synchronous grade 2 papillary transitional cell carcinoma of the bladder	Local resection	None (1y)
*Kostakopolulos* et al. *(1988)* [42]	66	M	Haematuria, renal colic	Left U	NS	None	Partial resection	None (6 months)
*Garritano* et al. *(1988)* [43]	49	M	Haematuria	Left middle U	Pedunculated lobulated tumour/3	None	Local resection	None (5 ys)
*Aubert* et al. *(1988)* [17]	71	M	Haematuria, flank pain	Right lower U	NS	None	Partial resection	None (5 ys)
*Page* et al. *(1991)* [44]	56	M	Haematuria	Distal U, bilateral	Multiple sessile lesions/right side lesion: 3 cm; 2 lesions of the left side: 2 cm each)	None	Right side: partial Uectomy; Left side: complete Uectomy	NS
*Kunimi* et al. *(1994)* [45]	42	M	Flank pain	Left middle U	Pedunculated polyp/ 2.7	Superficial transitional cell carcinoma grade 2 of the bladder (23 months later)	NephroUectomy	None (20 months after the diagnosis of carcinoma)
*de Knijff* et al. *(1997)* [46]	63	M	Urinary frequency and urge	Right distal U	NS/2	Invasive bladder tumour six years later, treated with cystoprostatectomy	Local resection	None
*Hoekx* et al. *(1998)* [47]	71	M	Haematuria, flank pain	Left distal U and right distal U	Smooth surface/NS	Synchronous grade 2 transitional cell carcinoma of the bladder (T1N0M0)	Left partial resection and right nephroUectomy	Multiple recurrences of urinary badder carcinoma (duration of follow-up NS)
*Lyon* et al. *(2006)* [48]	59	M	Haematuria	Left proximal U	Sessile lesion/2.5	None	Local resection	None (1 y)
*Kilciler* et al. *(2008)* [49]	62	M	Haematuria, flank pain	Middle U (side NS)	NS/2	None	NephroUectomy	None (NS)
*Mertziotis* et al. *(2012)* [50]	62	M	Haematuria, flank pain	Right upper U	Exophytic lesion/4	None	Nephrouretectomy	None (14 months)
*Murtaza* et al. *(2012)* [51]	35	M	Flank pain	Left distal U	Multiple small to large polypoid lesions	None	Local resection	None (6 months)
*Lopez-Fontana* et al. *(2012)* [52]	30	M	Haematuria	Right distal U	Polypoid lesion/1.6	None	Partial Uectomy	None (4 months)
*Luo* et al. *(2012)* [26]	70	M	Haematuria	Right U	Pedunculated	None	NephroUectomy	None (NS)
*Luo* et al. *(2012)* [26]	61	M	Flank Pain	Left U	Pedunculated	Not specified	Partial Uctomy	None (NS)
*Luo* et al. *(2012)* [26]	67	M	Asymptomatic	Left U	Multiple lesions/ Pedunculated	None	NephroUectomy	None (NS)
*Luo* et al. *(2012)* [26]	67	M	Haematuria	Left U	Multiple lesions/ Pedunculated	None	Local resection	None (NS)
*Luo* et al. *(2012)* [26]	73	M	Haematuria	Left U	Pedunculated	Not specified	Partial Uctomy	None (NS)
*Luo* et al. *(2012)* [26]	68	M	Haematuria	Left U	Pedunculated	Not specified	Partial Uctomy	None (NS)

Histological diagnosis of IUP can be difficult and several pathological conditions may enter differential diagnosis, including other urothelial neoplasms with endophytic growth patterns (i.e. papillary urothelial neoplasm of low malignant potential, low- and high-grade urothelial carcinoma), nested urothelial carcinoma, paraganglioma, carcinoid tumour, florid von Brunn nest proliferation and cystitis cystica et glandularis. Most of the investigated immunohistochemical markers are of little use in routine practice, and microscopic assessment remains the current gold standard. IUPs are benign tumours and can be successfully treated by conservative surgery. While specific molecular alterations are well described for papillary urothelial neoplasms, only few studies have been conducted on inverted lesions, suggesting a correlation between inverted growth and mismatch repair deficiency in urothelial carcinoma of the upper urinary tract [53].

Two additional cases of polypoid IUP of the renal pelvis and the ureter are herein presented with a systematic review of the literature.

## Clinical cases

### Case 1

A 76-year-old woman was admitted with persistent right flank pain and macroscopic haematuria. A computed tomography (CT) scan revealed a 2-cm polypoid lesion in the right renal pelvis, causing mild proximal hydronephrosis. The patient was otherwise in good health and advised to undergo nephroureterectomy due to the high likelihood of malignancy. Postoperative course was unremarkable, and the patient was discharged eight days after admission. She is alive and free of disease six years after treatment.

### Case 2

A 56-year-old man presented with gross haematuria. A CT scan demonstrated a filling defect in the lower third of the right ureter with no evidence of lithiasis. Owing to the distal location of the lesion, segmentary ureterectomy was performed. The patient is asymptomatic five years after complete excision of the tumour.

## Pathological findings

**Fig. 1:**
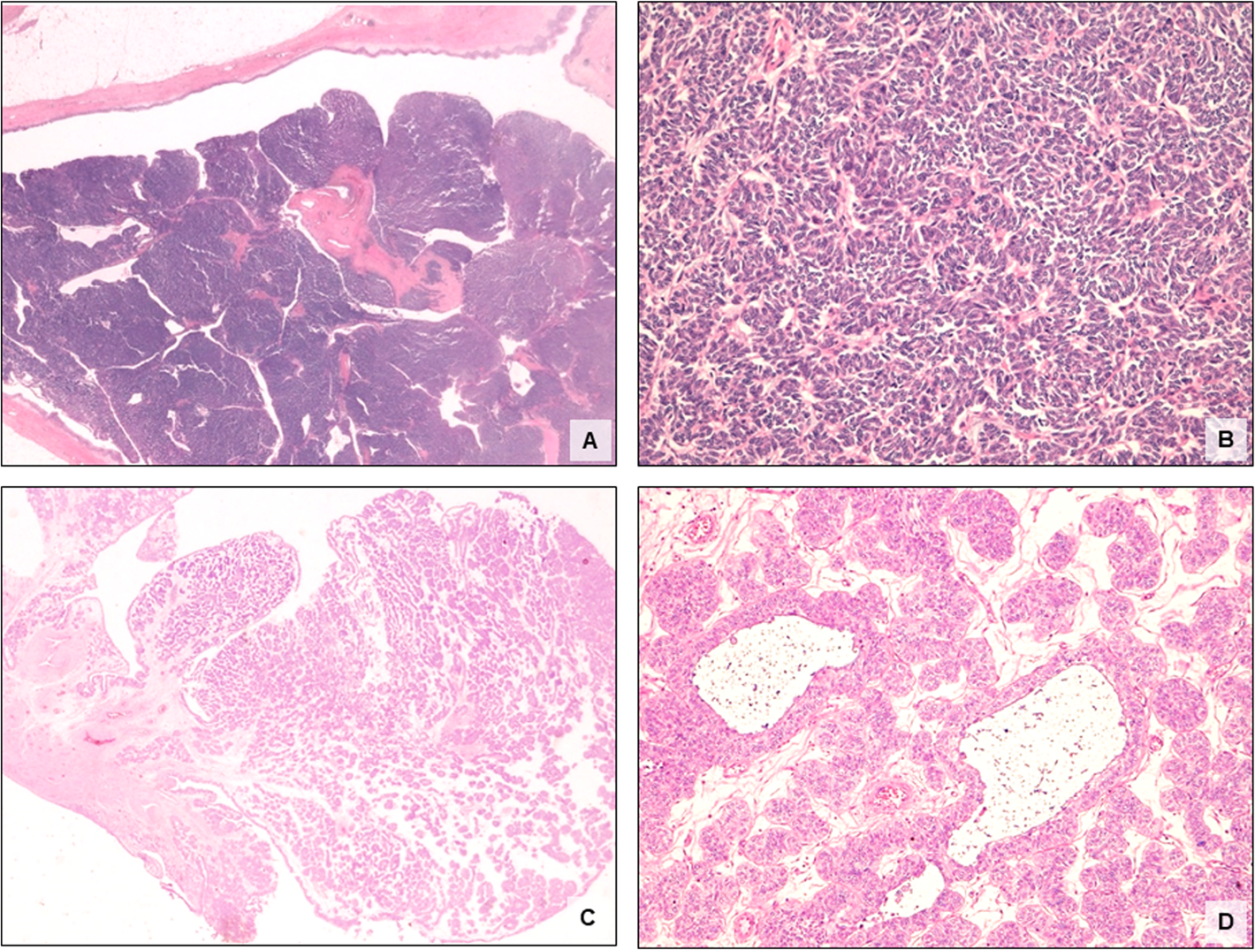
Histological features of two cases of IUP of the upper urinary tract. Sessile polypoid tumour of the renal pelvis consisting of anastomosing trabeculae and cords growing downward into the lamina propria, with prominent peripheral palisading in the trabeculae **(**Case 1: **a, b).** Pedunculated polypoid IUP of the distal ureter characterized by microcyst formation and foci of squamous metaplasia **(**Case 2: **c, d)**

Both cases displayed similar gross and histological features. In case 1, a sessile polypoid tumour measured 2 cm in greatest diameter. Case 2 presented as a 1.4-cm polypoid mass with a thin stalk. Microscopically, both lesions consisted of anastomosing trabeculae and cords growing downward into the lamina propria and lacked any true exophytic papillary component. Prominent peripheral palisading was seen in the trabeculae. There was no evidence of significant nuclear atypia and less than 1/10 high-power field mitotic figures were found. Hyalinised collagenous stroma was seen in case 1. Microcyst formation and foci of squamous metaplasia were occasionally observed in case 2. Histology was consistent with IUP (Fig. 1).

Representative sections of the lesions were selected for immunohistochemical analysis. As primary antibodies, we used rabbit monoclonal Ki-67 (clone 30.9, ready to use; Ventana, Tucson, AZ), rabbit monoclonal CK20 (clone SP33, ready to use; Ventana), mouse monoclonal PMS2 (clone A16–4, ready to use; Ventana), mouse monoclonal MLH1 (clone M1, ready to use; Ventana), mouse monoclonal MSH2 (clone G219–1129, ready to use; Ventana) and rabbit monoclonal MSH6 (clone SP93, ready to use; Ventana). Sections were stained on a Ventana BenchMark ULTRA immunostainer (Ventana Medical Systems). The procedure involved pretreatment with Cell Conditioning 1 followed by antibody incubation. The signal was then developed with ultraView Universal DAB Detection Kit for antibodies against Ki-67 and CK20. OptiView DAB IHC Detection Kit was employed for all other antibodies.

**Fig. 2:**
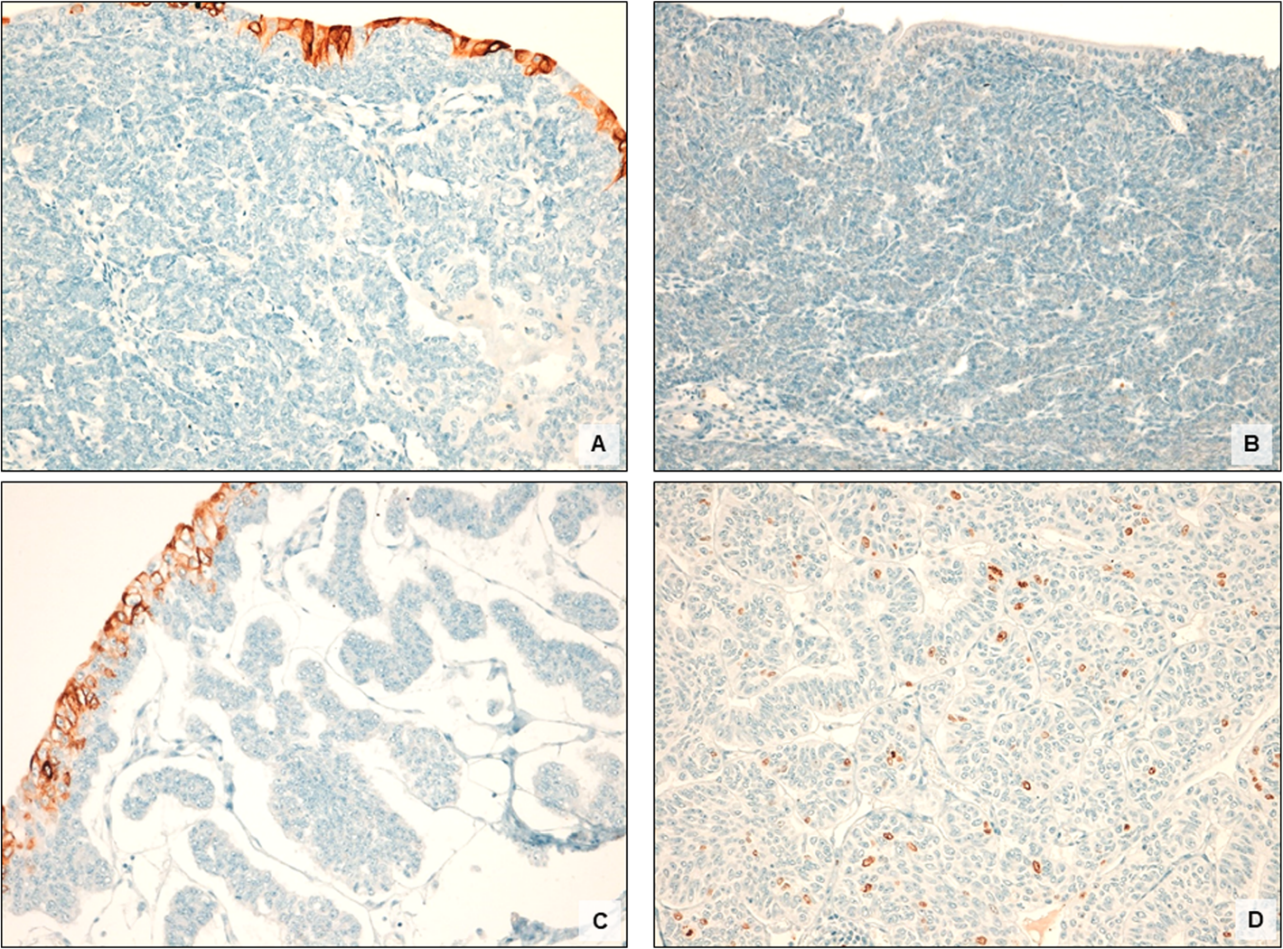
Immunohistochemical results in two cases of IUP pf the upper urinary tract. Both cases were negative for CK20 immunostaining **(**Case 1: **a**; Case 2: **c)** and showed low Ki-67 labelling index (< 1%) **(**Case 2: **b**; Case 2: **d)**

**Fig. 3:**
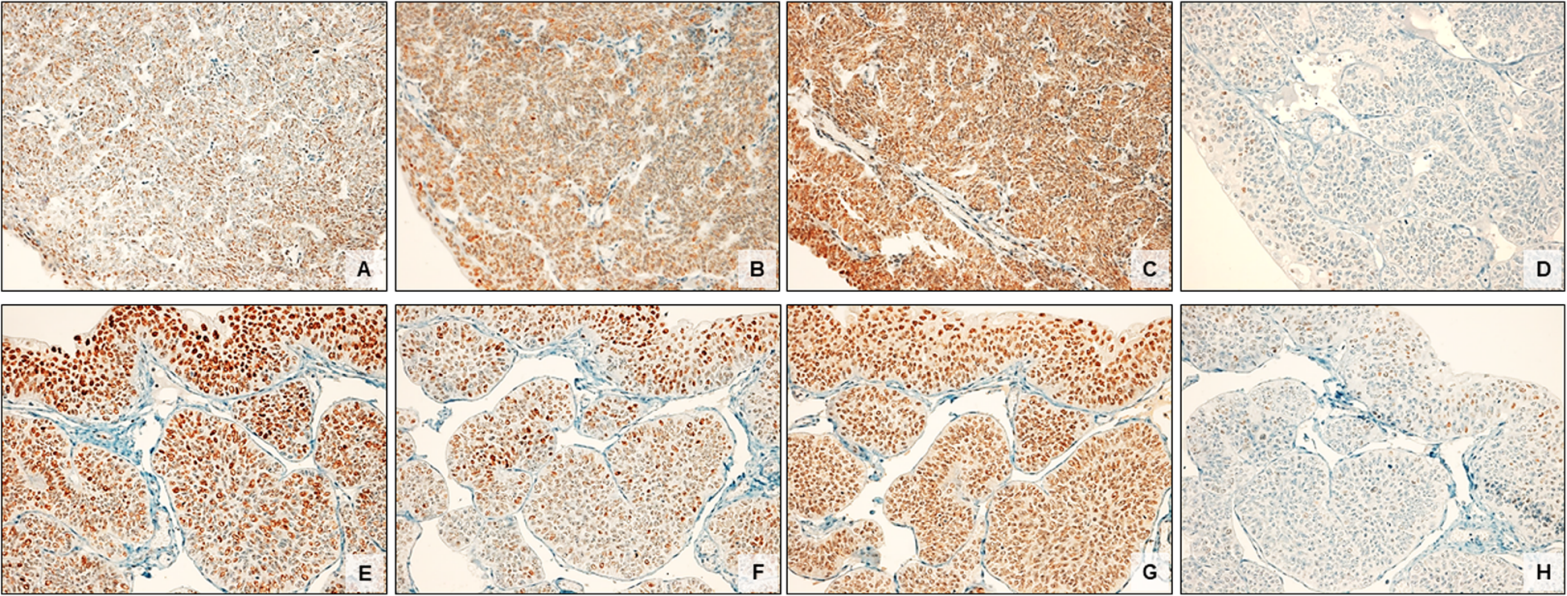
Expression of the mismatch-repair proteins in two cases of IUP of the upper urinary tract **(**Case 1: **a-d**; Case 2: **e-h).** Nuclear staining for MLH1 (**a, e**), MSH2 (**b, f**), PMS2 (**c, g**) was observed in both cases, whereas the tumours showed loss of MSH6 expression (**d, h**)

Both lesions were negative for CK20 and exhibited uniformly low Ki-67 (< 1%) (Fig. 2). Expression of the mismatch-repair protein was considered positive if at least 10% of neoplastic cells showed nuclear staining [54]. Loss of MSH6 was seen in both cases, alongside with retention of MLH1, MSH2, and PMS2 expression (Fig. 3).

## Discussion

IUP of the upper urinary tract is a benign tumour with 68 cases described to date in the English literature. It usually manifests in middle-aged adults within the 6th or 7th decade of life, and males are more commonly affected than females [26].

The most frequent presenting symptoms are haematuria, macroscopic or microscopic, and renal colic. Irritative symptoms, as well as urinary tract obstruction, have also been reported [55]. In a high percentage of cases, however, tumours are asymptomatic and detected during unrelated clinical investigations.

Preoperative diagnosis of IUP is difficult. Imaging studies may reveal non-specific findings such as filling defects of obstructive masses, often associated with hydronephrosis, hydroureter or renal stones [56]. Cytological morphology falls within the range of normal or mild atypia since IUP is covered by a normal and intact mucosal layer. Accurate preoperative diagnosis requires biopsy and visualisation through endoscopic examination. These procedures also provide therapeutic indications, thus avoiding unnecessary nephroureterectomy [26]. Due to the high likelihood of malignancy, preoperative biopsies were not carried out in our cases and patients underwent radical surgery.

Grossly, IUP presents as a solid or polypoid mass with smooth mucosal, non-papillary covering surface. Most tumours measure less than 3 cm in diameter but can reach up to 8 cm or more. They usually occur as solitary lesions, although 3.6–6% are bilateral or multicentric [55].

Histologically, IUP is characterised by endophytic growth of epithelial elements arranged in nests and cords, growing down from the surface urothelium into the lamina propria with expansible borders. Cystic areas and foci of squamous metaplasia are common. Neither fibrovascular cores nor desmoplasia are seen in IUP and stromal inflammation is minimal. Necrosis and mitotic activity are absent. Distinction between inverted papilloma and urothelial carcinoma with an endophytic growth pattern can be challenging. Contrary to IUP, urothelial carcinoma with inverted configuration shows cytological atypia, mitoses, nuclear pleomorphism and often displays an exophytic papillary component. In addition, invasion into the muscularis propria may occur in urothelial carcinoma but not in IUP. When biopsies are of small size or morphological artefacts and tangential sectioning obscure the lesion, differentiating between these biologically different entities becomes increasingly difficult [57].

Recently, Wobker et al. described 13 cases of a unique urothelial tumour occurring exclusively in the renal pelvis and ureter, named polypoid urothelial proliferation with inverted growth pattern (PUTIP). Morphologically, PUTIP exhibits hybrid features between a totally inverted PUNLMP, IUP and florid proliferation of von Brunn nests [58]. PUTIP may show a distinct inverted papilloma–like component with densely hyalinised collagenous stroma, but lacks the thin anastomosing cords typical of IUP.

In the present study, we observed low Ki-67 proliferation index and negativity for CK20 in both cases. A number of immunohistochemical markers have been shown to be frequently expressed in urothelial carcinomas, including the proliferation marker Ki-67 and CK20 [59]. IUP may be aneuploid and demonstrate high proliferative activity, although these features do not necessarily correlate with malignant behaviour [60, 61].

Our cases showed loss of MSH6 by immunohistochemistry, whereas expression of MSH2, MLH1 and PMS2 was retained. The molecular genetic abnormalities of IUP appear to differ from those of urothelial carcinoma, suggesting that these two neoplasms are unrelated [62]. Inverted-type urothelial carcinomas of the renal pelvis can be associated with MSI. Hartmann and co-authors examined 132 urothelial carcinomas of the upper urinary tract exhibiting some degree of inverted growth, and found that 35 (26.5%) were microsatellite unstable by polymerase chain reaction analysis [53]. Similar results were obtained by Harper in 214 patients with upper tract urothelial carcinoma tested for mismatch repair protein loss by immunohistochemistry [63]. In a multicentric study conducted on 62 IUPs of the urinary bladder Eiber and co-authors demonstrated aberrant immunostaining for MSH2 (5.8%), MLH1 (11.8%) and MSH6 (3.8%) [62]. As previously described, cellular loss of one MMR protein is not sufficient to cause detectable microsatellite defects [64]. Therefore, our observation may be spurious and unrelated to microsatellite instability, and should be confirmed in a larger series of IUPs of the upper urinary tract. In addition, our patients did not show any stigmata of Lynch syndrome or HNPCC-associated background.

Regarding treatment options, nephroureterectomy, local resection or partial ureterectomy with preservation of the kidney, and endoscopic surgery may be of use [65]. After excision, some authors recommend a follow-up protocol (endoscopy and radiographical studies) similar to that used in patients with low-grade urothelial carcinoma [26], while others do not advocate this rigorous and long-term follow-up due to the low risk of recurrence and favourable prognosis of IUP [66].

In conclusion, IUP of the upper urinary tract is an extremely rare tumour characterised by an inverted pattern of growth and constituted by normal to minimally atypical proliferating urothelium. The absence of progression of IUP on long-term follow-up argues against the need of patients’ continuous surveillance when strict diagnostic criteria are followed, a complete resection can be ascertained and no history of previous or concurrent urothelial malignancies is recorded.

## Data Availability

The authors confirm that the data supporting the findings of this study are available within the article.

## Abbreviations

IUP: Inverted Urothelial Papilloma
CT: Computed Tomography
PUTIP: Polypoid Urothelial Proliferation with Inverted Growth Pattern
RP: Renal Pelvis
NS: Not Stated
NA: Not Applicable
